# Advertising Doctors

**Published:** 1852-05

**Authors:** 


*Advertising Doctors.—Extract from the code of Ethics adopted by the National Medical Association.*

"It is derogatory to the dignity of the profession, to resort to *public advertisements, or private cards and hand-bills,* inviting the attention of individuals affected with particular diseases,—*pnblicly offering advice and medicine to the poor gratis,*" *&c.*

The foregoing extract is cited in view of the fact which is notorious, that the Faculty of the University of the city of New York has long been in the habit of these precise "practices of empirics," which are declared by the same code to be "highly reprehensible in a regular physician."

Having long since rebuked these unprofessional devices of quackery, for such they are, in however high places they are adopted, our attention has been renewedly called to the subject by the recent advertisement of *Professor Valentine Mott,* in the penny and two-penny papers of the city, "publicly offering advice and medicine to the poor gratis" in open violation of the above ethical rule, under the cover of the "college clinique" at the University in 14th street.

Our readers are aware of our exalted estimate of Dr. Mott, so often expressed; nor are our opinions changed as to his character or ability, either as a teacher or practitioner. But the higher the position he has ever held in our esteem, the greater our regret and humiliation to find his name associated with aught that is "derogatory to the dignity of the profession." Grant that it is the fruit of his recent renewal of associations with the men who have repented of having "eaten the oyster and thrown away the shell;" yet he should not soil his professional ermine, by consenting to any course unworthy of himself, and declared by our highest medical authority to be "highly reprehensible in a regular physician."

The influence of his example in this particular can not fail to enlist a host of imitators, who may deluge the newspapers with similar advertisements with impunity. For if "college cliniques" and those who hold them, may thus trample on the code of ethics, and our "Academy of Medicine" fail to discipline the offenders, by what rule is any member of the profession to be shunned and ostracised as a quack, and even refused admission into the "Academy" who commits no other offence than to advertise the clinique held in his office, and with the same stale trick of "Advice to the poor gratis?" We pause for a reply;—for we hold that no privileges of exemption from established ethical rules can be justly claimed by *professors,* to which every other member of the profession is not equally entitled.

Our neighbor of the Scalpel, a pupil of Dr. Mott, has been denounced and proscribed for years past, no other offence having been alleged than that of following in the footsteps of his illustrious master, by becoming what has been opprobriously termed an "advertising doctor." The indignation of the whole medical fraternity has been invoked against a "certain college" for having conferred the honors of the profession upon an individual in this city, because of his alleged "advertisements" to treat a certain class of patients, which were never a whit more exceptionable than the advertisement now appearing by Dr. Mott. And though, like every body else in the profession, he doubtless treated the "poor gratis," yet even *he* refrained from "publicly offering" to do so by advertisements in the Herald.

We could name an individual Oculist of this city, of sterling worth as a man, and acknowledged ability of a high order in his protession, who because he announced himself on his arrival in the country, by advertisements in the secular papers, setting forth his lectures, his cliniques and his "operations on the poor gratis," has been persecuted, and even insulted by professional dignitaries, until his sensitive nature, and modest merit had to be developed in the shade of that Coventry to which he had been consigned, and yet he has long since constrained the respect of the very men who proscribed him, by his reputation and success. Dr. W. will bear with us for giving his initials, because we wish to be understood.

To speak plainly, as is our wont, the profession generally, here and elsewhere, are becoming sensitive on the subject of these "advertisements" and other ethical improprieties, endured when committed by Professors, and punished among what some are pleased to call, the laity of the profession. For example, wherein consists the difference between the "advertisements" of the following *M. D.'s* except that they hail from different colleges. They all equally violate the ethical rule above cited by advertising; and as to publicly offering advice to the poor gratis, this is declared to be a double offence.—*New York Medical Gazette.*

